# Does youth-friendly mental health care improve therapeutic engagement and psychosocial outcomes?

**DOI:** 10.1177/10398562251351445

**Published:** 2025-07-01

**Authors:** Stephen Allison, Tarun Bastiampillai, Steve Kisely, Jeffrey CL Looi

**Affiliations:** College of Medicine and Public Health, 1065 Flinders University, Adelaide, SA, Australia; Consortium of Australian-Academic Psychiatrists for Independent Policy and Research Analysis (CAPIPRA), Canberra, ACT, Australia; Department of Psychiatry, 2541 Monash University, Clayton, VIC, Australia; College of Medicine and Public Health, 1065 Flinders University, Adelaide, SA, Australia; Consortium of Australian-Academic Psychiatrists for Independent Policy and Research Analysis (CAPIPRA), Canberra, ACT, Australia; School of Medicine, Princess Alexandra Hospital, 1974 The University of Queensland, Brisbane, QLD, Australia; Consortium of Australian-Academic Psychiatrists for Independent Policy and Research Analysis (CAPIPRA), Canberra, ACT, Australia; Academic Unit of Psychiatry and Addiction Medicine, Canberra Hospital, The Australian National University School of Medicine and Psychology, Canberra, ACT, Australia; Consortium of Australian-Academic Psychiatrists for Independent Policy and Research Analysis (CAPIPRA), Canberra, ACT, Australia

**Keywords:** youth-friendly, youth mental health, Australian headspace, dropout rates, psychotherapy attrition

## Abstract

**Objectives:**

We discuss the paradox of young people dropping out of the Australian Government national youth psychotherapy programme (headspace), which is co-designed by youth people.

**Conclusions:**

A very large percentage of young people drop out of psychotherapy before completing evidence-based treatment. Youth-friendly psychotherapy services are hypothesised to improve therapeutic engagement and psychosocial outcomes. However, empowered young people may not choose greater engagement with psychotherapy. For example, the Australian Government recognises the right to youth-friendly services and headspace emphasises providing young people with access to support where, when, and how they want. Most appear to want very short courses of psychotherapy (1–3 sessions), which are associated with lower than expected psychosocial outcomes compared to other real-world services. Only the 20% who engage in 6 or more sessions have outcomes comparable to other psychotherapies. These findings have international significance because similar youth-friendly psychotherapy programmes are being established around the world.

Young people should be heard, given that involving them in service development, design and delivery helps to improve access and increases engagement in and satisfaction with services. (Eline Wittevronge and colleagues, 2023)^1^

This perspectives article provides a critical examination of youth-friendly care. It is generally assumed that involving young people in the design and delivery of mental health services improves therapeutic engagement and psychosocial outcomes.^1^ However, there have been few empirical studies of the impact of youth-friendly care.^2^ We therefore investigate whether the Australian Government national youth psychotherapy programme (headspace) improves engagement and outcomes when compared to other real-world psychotherapy services.^3–5^

High dropout rates are a threat to the effectiveness of youth psychotherapy in real-world settings. A very large percentage of young people drop out before completing evidence-based psychotherapies such as cognitive behavioural therapy. While the average number of sessions in randomised trials is around 16, the average number of sessions attended by young people is only 3-4 in real-world settings with the mode being as low as 1-2 sessions.^3^ These incomplete courses of psychotherapy are unlikely to achieve optimal psychosocial outcomes.

## Australian Government youth-friendly psychotherapy services

Youth-friendly mental health services are hypothesised to improve engagement in evidence-based psychotherapy, providing early intervention that changes the course of youth-onset common mental health disorders.^4,5^ We focus on youth-friendly services in the independently evaluated Australian Government headspace programme.^4,5^ Government has funded several evaluations of headspace, most recently by a consortium of Klynveld Peat Marwick Goerdeler (KPMG), the Social Policy Research Centre at the University of New South Wales, and the not-for-profit organisation, batyr.^6^ Evaluations of headspace have also been published in the peer-reviewed literature.^7–9^

These evaluations rely on routine data collection by a national network of 164 standalone headspace youth centres.^5^ These centres have delivered more than 6.7 million government-funded services to over 865,000 young Australians since establishment in 2006.^8^ Therapists complete questionnaires at each session about the young person’s level of functioning, the need for further therapy, and referral to other services.^6^ Young people are also invited to complete the Kessler Psychological Distress Scale (K-10) after each session and at 90 days post the final session, although completion rates at 90-day follow-up are very low (4.6%).^6^

The provision of psychotherapies is the predominant treatment approach in headspace including cognitive behavioural therapy, supportive counselling, acceptance and commitment therapy, and a variety of other psychotherapeutic interventions.^7^ Additionally, alcohol and drug counselling, physical and sexual health, and vocational support make up a very small proportion of headspace services.^6^

The Australian Government recognises the right to youth-friendly services and headspace meets the World Health Organization’s standards (Box 1).^10^ These are further defined as services being ‘accessible, appealing, flexible, confidential and integrated, where youth feel respected, valued, and welcome to express themselves authentically, without discrimination of any kind; it is a developmentally and culturally appropriate service that mandates youth participation in service design and delivery, to empower youth and help them gain control over their lives’.^2^

**Box 1: Youth-friendly mental health services**

World Health Organization

• Accessible: Young people can obtain the mental health services that are available.
• Acceptable: Young people are willing to obtain the mental health services that are available.
• Equitable: All young people, not just selected groups, are able to obtain the mental health services that are available.
• Appropriate: The right mental health services (i.e. the ones they need) are provided to them.
• Effective: The right mental health services are provided in the right way, and make a positive contribution to their mental health.

Australian Government

• Ensuring that young people can access the mental health care they need in an appropriate, accessible, and youth-friendly way by providing accessible, welcome, inclusive, and non-stigmatising services
• Providing an appropriate service approach for young people with mild to moderate, high-prevalence mental health conditions
• Providing culturally appropriate and inclusive services
• Enabling young people to access support where, when, and how they want
• Participation of young people in the design and delivery of headspace
• Improving mental health and clinical outcomes for young people.

headspace has a ‘no wrong door’ approach for easy access, so that no young person is turned away.^4,5^ This approach is effective in enabling young people to access support where, when, and how they want, although some challenges are acknowledged around open hours and waiting times.^6^ Reference groups ensure youth participation in headspace strategic planning, service development, delivery, and evaluation.^8^ headspace is proposed as an exemplar of youth-friendly care, and similar youth-friendly programmes are being established in numerous countries.^4,5^

## Youth engagement with Australian Government psychotherapy services

The first issue is whether headspace’s youth-friendly services are associated with low psychotherapy dropout rates, which we define as the premature discontinuation of psychotherapy prior to optimal psychosocial benefit.^11^ One method of measuring dropout rates is to pre-specify the number of sessions of psychotherapy that is likely to be effective.^12^ The average treatment dose in randomised trials of youth psychotherapy is approximately 16 sessions, so this could be the pre-selected number.^3^ However, this method misclassifies episodes where young people discontinue psychotherapy because of early recovery and also misclassifies those episodes with many sessions but little progress. A second method is to operationalise dropout rates as those treatment episodes that do not result in clinically significant change with discharge scores within the normal range on a selected symptom measure such as the K-10 at the final session.^12^

According to the KPMG headspace evaluation, most young people meet these two empirical definitions of dropping out by receiving very short sub-therapeutic episodes of psychotherapy that do not result in clinically significant change.^6^

**Figure 1.**
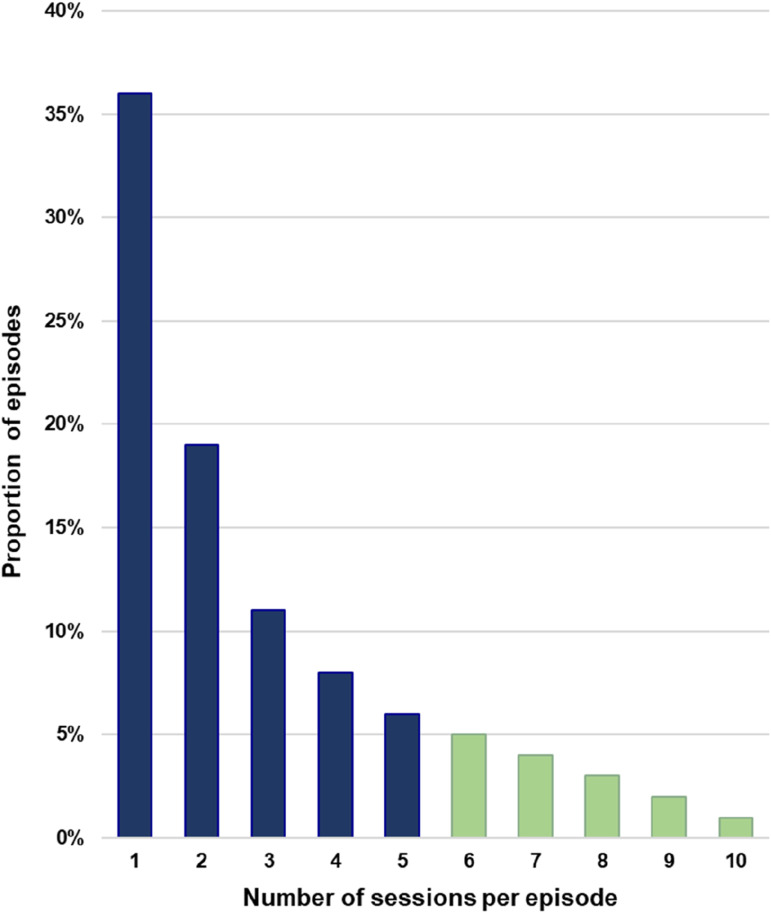
Benchmark of six or more sessions per episode in Australian Government headspace services. Data source: From the national headspace dataset as reported by a Commonwealth of Australia, Department of Health evaluation (2022), that was undertaken by a consortium of Klynveld Peat Marwick Goerdeler (KPMG), the University of NSW Social Policy Research Centre, and batyr.

Firstly, this is demonstrated by the distribution of sessions per episode, drawn from data on more than 60,000 headspace episodes (Figure 1).^6^ Around 36% of these episodes comprise a single session and two-thirds of episodes (66%) consist of only 1–3 sessions. This dropout rate is higher than a large study of real-world youth psychotherapy reporting a 42% dropout rate by the third session.^5^ Only 5% of headspace episodes consist of more than 10 sessions, which is the maximum number funded by the Australian Government.^6^ This in turn is lower than the average in randomised trials of around 16 sessions.^3^ This distribution of headspace sessions has been consistent, with no obvious improvement in treatment engagement over time.^6^

Secondly, these short headspace episodes do not usually result in clinically significant change, which is observed in only 17% of episodes as measured by the K-10.^6^ This is less than expected in real-world services.^6^ Average K-10 scores at the final session indicate that young people still have raised levels of psychological distress when they exit headspace.^6^ According to KPMG, ‘In general, headspace outcomes only become comparable to other psychotherapies once six or more sessions have been accessed’.^6^

In addition, KPMG did not identify any reliable area-level effects as a result of access to headspace such as reductions in youth hospitalisation rates.^6^ Moreover, the nationwide rollout of headspace centres did not prevent a rise in the prevalence of common mental disorders, deliberate self-harm, and youth suicide in Australia, according to another analysis by Jorm and Kitchener.^13^ This is an example of the ‘treatment-prevalence paradox’, wherein psychotherapy for common mental disorders becomes more widely available, but population prevalence does not fall.^14^

One possible explanation for these short episodes is that headspace aims to provide early intervention for mild psychological distress, so young people may only need a few sessions.^5^ However, the ‘no wrong door’ approach means that all levels of psychological distress are treated at headspace, and most young people exit while still needing treatment. In a study by Seidler and colleagues of a cohort of more than 80,000 young people, headspace therapists reported that most young people (70%) dropped out while requiring further psychotherapy.^9^ Another 10% were referred to other agencies, so only 20% of this cohort were ‘headspace therapy completers’, which may help explain the modest outcomes.

Given the main emphasis of headspace is allowing young people to access support where, when, and how they want, it appears that empowered young people may simply prefer brief therapy as observed in other real-world settings.^3^ Various other factors may contribute to a young person’s decision to drop out including travel time to the centre or waiting periods.^6^ Young people are more likely to drop out if psychotherapy is not helping them.^8^ They may not like regular appointments, missing leisure activities, or talking about distressing topics.^15^ Some may experience stigmatisation from peers because they are attending a mental health service.^16^ They could encounter the common adverse effects of youth psychotherapy such as unpleasant memories.^17^ Others may decide that psychotherapy does not adequately address real-world concerns such as poverty, unemployment, or family violence.^15^

Certain demographic groups are more likely to drop out of headspace including those who are older (18–25 years), male, Aboriginal or Torres Strait Islander, and/or those living in a rural location.^9^ To promote engagement, some Aboriginal and Torres Strait Islander young people suggested that headspace therapists could be more culturally competent.^6^ Young people from culturally and linguistically diverse backgrounds would also like more cultural diversity amongst therapists.^6^

KPMG recommends that headspace should assertively support young people so they receive 6 or more sessions (Box 2).^6^ Therefore, the proportion of episodes with at least 6 sessions could become a key performance indicator, although only 20% of headspace episodes currently meet this benchmark (Figure 1). Suggested headspace programme improvements are targeted protocols for groups at higher risk of dropping out and the discussion of young people’s session-by-session experiences.^8,9^

If implemented systematically, these measures could have small favourable effects on dropout rates and psychosocial outcomes.^18^ To follow these potential improvements, headspace outcomes should be routinely reported by the Australian Government including waiting times, missed appointments, the mean number of psychotherapy sessions, and rates of clinically significant improvement, as the national government-funded ‘Talking Therapies’ programme does in the United Kingdom.^19^ Reporting could include data from individual headspace centres because there may be considerable regional variation.

## Limitations and further research

There are a number of limitations in these evaluations of headspace.^6,7,9^ Most notably, they rely on routinely collected data across a large number of centres with differing clinical governance. Data compliance by young people is very low at 90 days after exiting headspace. It is therefore not possible to evaluate whether dropping out is associated with poorer clinical outcomes. However, K-10 scores are often in the severe range at the final session, so dropping out does not augur well.^9^ Despite these data constraints, headspace evaluations provide valuable insights into a national youth-friendly psychotherapy programme.

**Box 2: KPMG recommendations for Australian Government headspace services**

Support longer episodes of care

headspace outcomes only become comparable to other psychotherapies once six or more sessions have been accessed. Given that psychosocial outcomes are strongly associated with engagement and treatment through headspace, there are opportunities to improve user experience and clinical governance arrangements to support longer episodes of care for young people.

Non-integrated youth services

Alcohol and other drugs, physical and sexual health, and vocational support represent a very low proportion of headspace services provided. Stakeholder feedback has suggested this may not reflect local or regional need. For example, a region with significant substance misuse issues amongst young people may need a greater mix of alcohol and other drugs services at the local headspace.

Gaps in data collection

Filling gaps in headspace data could support better monitoring and evaluation of outcomes, for example,

• The reason for ending psychotherapy to differentiate between planned exits and unplanned exits
• Outcomes beyond 90 days after psychotherapy with a particular focus on episodes involving a single session
• Referral data on the stage of care at the point of referral (e.g. intake, mid-treatment, and exit), and whether the referral onwards was taken up.

Data linkage studies

The longer-term impacts of headspace are not measured. headspace data should be collected in a way that allows it to be linked to other datasets, so that outcomes over time of young people who access headspace can be better understood when compared to those who do not access headspace.

Experimental studies

So that young people’s outcomes can be rigorously measured and attributed to headspace, the Australian Government should fund experimental studies, for example, research into the effectiveness of headspace single sessions, given that approximately 36% of episodes of care have a single session.

Report full costs

headspace services do not currently collect or report the full costs of operation, so cost-effectiveness can only be estimated. The Australian Government should prioritise the collection of full cost information from headspace centres.

KPMG recommends data linkage studies to assess the impact of headspace early intervention on subsequent community service use, self-harm and substance abuse hospitalisations, employment outcomes, and suicide deaths compared to those who do not access headspace (Box 2).^6^ headspace is hypothesised to have better outcomes than general practice and child and adolescent mental health services but comparison studies are required to demonstrate this superiority.^5^

Studies with experimental designs are also recommended by KPMG.^6^ Pre-post studies may show positive findings for Australian community youth mental health services, but when controlled designs are used, studies do not usually yield significant results.^20^ So experimental designs are required to determine whether headspace interventions are more effective than other active treatments. Any adverse effects of headspace should also be comprehensively measured and reported because early intervention may be associated with labelling, stigmatisation, and negative experiences of youth psychotherapy.^16,17^

## Conclusions

This critical evaluation of youth-friendly care found evidence that the Australian Government youth psychotherapy programme has empowered young people to access support where, when, and how they want. Under these conditions, it appears that the vast majority of young people want short courses of psychotherapy, which runs counter to the hypothesis that youth-friendly care improves engagement in evidence-based psychotherapy and thereby changes the course of youth-onset mental disorders. Instead, empowered young people appear to prefer brief therapy. Without active parental and professional encouragement, they seem to choose less demanding options rather than optimal treatment. These findings have global implications because similar youth-friendly psychotherapy programmes are being established around the world and their impact may be lessened by high dropout rates from evidence-based psychotherapy and sub-therapeutic outcomes.

## References

[1] Wittevrongel E van Winkel R Jackers M , et al. How to make mental health services more youth‐friendly? a Delphi study involving young adults, parents and professionals. Health Expect 2023; 26: 2532–2548. 37608557 10.1111/hex.13832 PMC10632649

[2] Hawke LD Mehra K Settipani C , et al. What makes mental health and substance use services youth friendly? a scoping review of literature. BMC Health Serv Res 2019; 19: 257. 31029109 10.1186/s12913-019-4066-5 PMC6486969

[3] Weisz JR Venturo-Conerly KE Fitzpatrick OM , et al. What four decades of meta-analysis have taught us about youth psychotherapy and the science of research synthesis. Annu Rev Clin Psychol 2023; 19: 79–105. 36750262 10.1146/annurev-clinpsy-080921-082920

[4] McGorry P Mei C Chanen A , et al. Designing and scaling up integrated youth mental health care. World Psychiatry 2022; 21: 61–76. 35015367 10.1002/wps.20938 PMC8751571

[5] McGorry PD Mei C Dalal N , et al. The Lancet Psychiatry Commission on youth mental health. Lancet Psychiatry 2024; 11: 731–774. 39147461 10.1016/S2215-0366(24)00163-9

[6] Commonwealth of Australia . Final report: evaluation of the national headspace program from Klynveld Peat Marwick goerdeler (KPMG), the university of NSW social policy research centre, and batyr, Canberra, Australia. Department of Health, Commonwealth of Australia, 2022.

[7] Rickwood D McEachran J Saw A , et al. Sixteen years of innovation in youth mental healthcare: outcomes for young people attending Australia’s headspace centre services. PLoS One 2023; 18: e0282040. 37390108 10.1371/journal.pone.0282040 PMC10313045

[8] Rickwood D Albrecht S Telford N . Young people's participation in their own mental health care: session-by-session feedback in youth mental health services (headspace). Early Interv Psychiatry 2025; 19: e13600. 39080916 10.1111/eip.13600 PMC11730746

[9] Seidler ZE Rice SM Dhillon HM , et al. Patterns of youth mental health service use and discontinuation: population data from Australia’s headspace model of care. Psychiatr Serv 2020; 71: 1104–1113. 32790590 10.1176/appi.ps.201900491

[10] World Health Organization . Making health services adolescent friendly: developing national quality standards for adolescent friendly services. Geneva, Switzerland: World Health Organization, 2012.

[11] Roseborough DJ McLeod JT Wright FI . Attrition in therapy: a survival analysis. Res Soc Work Pract 2016; 26: 803–815.

[12] Swift JK Greenberg RP . Premature discontinuation in adult psychotherapy: a meta-analysis. J Consult Clin Psychol 2012; 80: 547–559. 22506792 10.1037/a0028226

[13] Jorm AF Kitchener BA . Increases in youth mental health services in Australia: have they had an impact on youth population mental health? Aust NZJ Psychiatry 2021; 55: 476–484. 10.1177/0004867420976861 33300364

[14] Ormel J Hollon SD Kessler RC , et al. More treatment but no less depression: the treatment-prevalence paradox. Clin Psychol Rev 2022; 91: 102111. 34959153 10.1016/j.cpr.2021.102111

[15] de Soet R Vermeiren RRJM Bansema CH , et al. Drop-out and ineffective treatment in youth with severe and enduring mental health problems: a systematic review. Eur Child Adolesc Psychiatr 2024; 33: 3305–3319. 10.1007/s00787-023-02182-z PMC11564352 36882638

[16] Galderisi S Appelbaum PS Gill N , et al. Ethical challenges in contemporary psychiatry: an overview and an appraisal of possible strategies and research needs. World Psychiatry 2024; 23: 364–386. 39279422 10.1002/wps.21230 PMC11403198

[17] Watson PN Leroux E Chowdhury M , et al. Unexpressed wishes and unmet needs: a mixed methods study of youth negative experiences in psychotherapy. J Child Fam Stud 2023; 32: 424–437.

[18] de Jong K Conijn JM Gallagher RAV , et al. Using progress feedback to improve outcomes and reduce drop-out, treatment duration, and deterioration: a multilevel meta-analysis. Clin Psychol Rev 2021; 85: 102002. 33721605 10.1016/j.cpr.2021.102002

[19] Clark DM Canvin L Green J , et al. Transparency about the outcomes of mental health services (IAPT approach): an analysis of public data. Lancet 2018; 391: 679–686. 29224931 10.1016/S0140-6736(17)32133-5 PMC5820411

[20] Savaglio M O’Donnell R Hatzikiriakidis K , et al. The impact of community mental health programs for Australian youth: a systematic review. Clin Child Fam Psychol Rev 2022; 25: 573–590. 35171386 10.1007/s10567-022-00384-6 PMC8853061

